# Factors Affecting Engagement With Portfolio-Based Reflection Among Internal Medicine Trainees: A Survey-Based Study

**DOI:** 10.7759/cureus.63022

**Published:** 2024-06-24

**Authors:** Jeorghino Lodge, Cornelius Neptune, Nwe Ni Tun

**Affiliations:** 1 Internal Medicine, Blackpool Victoria Hospital, Blackpool, GBR; 2 Acute Medicine, Wrightington, Wigan and Leigh NHS Foundation Trust, Wigan, GBR; 3 Internal Medicine, Blackpool Teaching Hospitals, Blackpool, GBR

**Keywords:** doctors, medicine, portfolio, barriers, engagement, reflection

## Abstract

Background

Contemporary medical education emphasizes that postgraduate clinicians should look at their daily experiences as an opportunity to learn and advance their knowledge and practice of medicine. This is the concept of reflective practice. Internal medicine trainees (IMT) in the UK are encouraged to record written reflections in their electronic portfolios but it is not a mandatory requirement.^ ^There is literature suggesting that the level of engagement with these written reflections is varied and that when these are produced, they can be superficial. Thus, the aim of this research was to ascertain what percentage of trainees engaged in written reflections and the factors that affected the likelihood they would reflect. There are no studies that have attempted to quantify de novo engagement with reflective practice and to quantify the significance of different theorized barriers to reflection.

Methods

This study was in the form of a quasi-experimental cross-sectional study. A 15-item survey was sent out to the IMT in the northwest deanery of England (n=592). The survey remained open for approximately three months with periodic reminders sent out to the trainees. The survey was closed to further responses when the number of responses reached the predetermined sample size of 240 (5% margin of error at a confidence interval of 95%). The data were analyzed by chi-square testing and represented using descriptive statistics.

Results

There were 243 responses to this survey. A total of 81.5% (n=198) wrote reflections in their portfolio and 19.5% (n=45) did not write any reflections. The main content of written reflections were clinical outcomes (positive and negative), teaching, and new learning. Several background factors had a statistically significant influence on the likelihood that trainees would write reflections in their portfolios. These included their stage of training, years practicing medicine, location of primary medical training, first exposure to reflective practice, and whether they have ever been tutored on reflection. Concerns about legal or General Medical Council (GMC) use of reflective notes against trainees also significantly impacted on reflection. The main perceived barriers to written reflections were the fact that trainees felt they had no time to properly reflect and the lack of perceived benefits from reflections.

Conclusion

Most trainees wrote reflections in their portfolios, but the majority did not perceive any benefits in doing this. The varied backgrounds of trainees seem to have an impact on their likelihood to reflect and strategies to increase engagement would need to address this.

## Introduction

Reflective practice in medicine refers to the process of analyzing experiences, actions, and decisions to improve clinical practice [1]. The concept has very early beginnings - in 1933 Dewy wrote about increasing reflection among learners to add meaning and relevance as well as promote growth [2]. Since then, reflection has become topical among medical educators with much written in the literature about the proposed benefits it can affect [3]. The overarching concept of reflection is that by deconstructing all our experiences, we can better inform our decisions when we are faced with similar situations. It is felt that reflection that is done meaningfully, rather than superficially, can enhance communication, increase knowledge of the relevant subject area, and overall improve patient safety [4]. These skills can all be crucial to clinicians and healthcare professionals more widely [5].

Reflection can take many different forms including self-reflection, group/peer reflection, and supervisory reflection - all of which can be formal or informal [6]. While these many different forms of reflection exist, the curriculum for internal medicine trainees (IMT) in the United Kingdom refers to the use of written reflections as evidence of the specialty's capabilities in practice. The curriculum emphasizes the need for frequent reflection and feedback on clinical encounters without further details on how this should be facilitated, whose responsibility it is to govern this, and whether reflections need assessing [7].

The literature supporting the benefits of reflective practice in postgraduate medical education is often based on qualitative research looking at different aspects of the concept and there is variation across different professions [8]. There is yet to be a unified guidance on applying reflective practice in healthcare education so that all healthcare workers can gain the most from the process. Here, we take a quantitative look at what barriers are important to consider in helping trainees unlock the theorized benefits of reflection. The potential benefits for clinicians who engage in reflection seem to be generally accepted by most in the field and as such it continues to be a heavily researched area. The main research focuses on the ongoing division on how to fit reflective practice into medical education, where is it best placed along the learning continuum and importantly there is a big focus on ways to assess reflective practice [3].

This examination of the literature led us to ask several questions within the local context but the most important of which was whether trainees were reflecting and, if they were, if they were experiencing any of the theorized benefits in their clinical practice. This is something lacking in the literature which seems to have a focus on assessing trainees on their ability to engage in reflection specifically within the confines of the research project, but not in the context within which they would normally be expected to reflect. With this study, we look at clinicians in a training program for internal medicine to ascertain the level of engagement with reflection as prescribed by their training curriculum.

There has been no study that has attempted to provide a quantification of the extent to which physicians are engaging in reflection. Moreover, there is no report within the literature on the quantitative effects of different factors on the likelihood that trainees would write reflections when they are recommended but not mandatory.

## Materials and methods

Study question and objective**

The primary objective of this study was to ascertain the level of engagement with written reflection in the online portfolio for IMT. Secondary objectives were to ascertain what factors affected whether trainees were likely to reflect, to understand the general content of written reflections, and to determine the main perceived barriers to reflection.

Study design

We conducted a quasi-experimental cross-sectional study utilizing a 15-item questionnaire.

Setting and participant

The survey was taken from IMT in the northwest deanery of the United Kingdom (n=592). We utilized a convenience, non-probabilistic sampling method for participant selection.

Questionnaire

One open-ended question asking about trainees’ understanding of the concept of reflective practice. Some questions had fixed choices asking about the stage of training, whether trainees have reflected and questions asking whether different factors affected their training. There were questions that combined fixed responses with an “other” option to allow trainees to expand on the location of training, introduction to reflection, the content of reflection, and the main perceived barrier to reflection. Two questions requiring numerical responses on years in practice and a number of reflections written. There was one 5-point scaled question asking trainees about the extent to which they agreed reflecting had improved their clinical practice.

The face and content validity of the questionnaire were ensured by pilot review with a group of five IMT. Their responses to the early version were scrutinized by each reviewer independently and suggested changes were made by unanimous agreement to arrive at the final version of the questionnaire. This has been presented in the appendices.

Sample size**

The Qualtrics online sample size calculator was used to estimate a representative sample. Using sample proportion at 50% it was estimated that a sample size of 234 would be needed for a margin of error of 5% and a confidence interval of 95%. The acceptable number of responses was taken as 240 to account for the possibility of incompletely filled surveys that would be discarded.

Data collection**

We opted for the user-friendly and accessible Google Forms tool for data collection. The link to the Google survey was conveniently sent to the trainees via the regional administrators in the form of an email. To ensure inclusivity, the same link was also shared with the trainees via local WhatsApp groups in their host hospitals. This approach enhanced visibility for those who did not frequently access emails. Regular emails and messages were dispatched to the trainees on a weekly basis to encourage their participation. Once the calculated sample size was reached, the survey was closed to further responses.

Bias

We implemented several methods to mitigate bias. The Google form used was formatted to accept only one reply from each IP address to prevent duplicate responses. Results were initially scrutinized by two researchers independently to determine if any questionnaires were incomplete or improperly completed. If so, these would be reviewed by all three researchers to determine if they would be excluded.

Data analysis 

All completed questionnaires were utilized as part of the data set. Continuous variables were represented through measures of central tendencies (median) and dispersion (interquartile range). For categorical variables, descriptive analysis was conducted using frequencies. For association between categorical variables, chi-squared testing was performed and p-values of <0.05 were taken as statistically significant. Chi-squared testing was performed using Excel software. Measurements of effect size were calculated from chi-squared values as Cramers' V to account for the difference in group size between those who wrote reflections and those who did not.

## Results

A total of 198(81.5%) trainees wrote reflections in their portfolios. Table 1 shows the number of reflections written and the main content of those reflective notes. The median number of reflections written increased as trainees moved further along the training program. The majority of the group wrote reflections on negative clinical outcomes (80.8%, n=160) or organized teaching (80.3%, n=159).

**Table 1:** Number and content of trainees’ reflective notes and perceived benefits N=total population *The numbers here represent the year in the training program. IMT1: trainee is in the first year.

Contents	Mean (IQR)
How many reflections have you written?	
IMT1*	4( 2-6)
IMT2*	20 (9-25)
IMT3*	25 (5-30)
What do you reflect on?	% (N=198)
Negative Clinical Outcome	80.8(160)
Organized teaching events	80.3(159)
Positive Clinical Outcome	70.2(139)
Any new learning on the job	57.6(114)
Other things	12.6(25)
Has your practice improved by reflecting?	% (N=198)
1 (Strongly disagree)	32.3(64)
2	9.6(19)
3	32.8(65)
4	22.2(44)
5 (Strongly agree)	3.0(6)

A much greater proportion (41.9%, n=83) of trainees who write reflections did not feel that this improved their clinical practice by scoring on the lower end of the 5-point scale when asked this question compared to those who felt that they have improved and scored 4/5 on the scale (25.3%, n=50). Table 2 compares how different factors affect whether or not trainees reflected. The two groups were considered as below for some background characteristics.

**Table 2:** Factors affecting likelihood trainees would write reflection p <0.05 suggest significance. IMT1: the trainee is in the first year, IMT2 means the trainee is in the second year *Cramers V values interpretation 0.1-0.2 Weak association, 0.2-0.4 moderate association, 0.4-0.6 relatively strong association, 0.6-0.8 strong association, 0.8-1.0 very strong

	Factors affecting reflection	Wrote reflection % (n)	Did not write reflection % (n)	P-values	V*
Training Year	IMT1	28.7 (57)	42.2 (19)	0.012	0.19
	IMT2	35.4 (70)	44.4 (20)		
	IMT3	35.9 (71)	13.3 (6)		
Years in Practice	1-5yrs	70.2 (139)	57.8 (26)	<0.001	0.146
	6-10yrs	26.8 (53)	42.2 (19)		
	>10yrs	3.0 (6)	0 (0)		
PMQ Location	UK	56.1 (111)	0 (0)	<0.001	0.687
	Europe	0 (0)	44.4 (20)		
	Asia	25.3 (50)	28.8 (13)		
	Middle East	9.6 (19)	13.3 (6)		
	Africa	9.1 (18)	13.3 (6)		
Exposure to RF	Medical school	59.1 (117)	15.6 (7)	<0.001	0.431
	UK Medical Practice	30.3 (60)	84.4 (38)		
	Overseas medical practice	10.6 (21)	0 (0)		
Training/Tutoring	Received	54.0 (107)	15.6 (7)	<0.001	0.3
	Not Received	46.0 (91)	84.4 (38)		
Supervision	Input	51.5 (102)	44.4 (20)	<0.001	0.055
	No Input	48.5 (96)	55.6 (25)		
Legal Considerations	Worried	54.5 (108)	57.8 (26)	<0.001	0.025
	Not Worried	45.5 (90)	42.2 (19)		
GMC Considerations	Worried	80.8 (160)	62.2 (28)	<0.001	0.173
	Not Worried	19.2 (38)	37.8 (17)		

Trainees in this group, who wrote reflections, tended to be in the later stages of training (71% (n=141) were IMT2 and IMT3). The majority, 70.2% (n=139), had between one to five years of experience. They mainly had primary medical training in the UK (56%, n=111). Fifty-nine percent (n=117) were first introduced to reflective practice in medical school and 54% (n=107) received training/tutoring on reflective practice. The majority of these trainees had topics for reflection suggested to them by their supervisors (51.5%, n=102).

A total of 45 (18.5%) respondents did not write any reflection. This group was mostly IMT1 and IMT2 (86.7%, n=39), that is, they are in the earlier stages of training. The majority of these trainees had one to five years of experience (57.8%, n=26) but a significant proportion also had six to 10 years of experience (42.2%, n=19). They have more experience in medical practice than those who wrote reflections. No trainees in this group completed primary medical education in the UK but in other parts of Europe (44.4%, n=26) and Asia (28.8%, n=13). Eighty-four percent (n=38) have never received any tutoring/training on reflective practice and only 44.4% (n=26) have had their supervisor suggest topics to reflect on following meetings.

In the majority of cases, the use of reflective notes for any legal purposes is a concern for trainees. This was true for those who have engaged in some reflection (54.5%, n=108) and those who have never written any reflection (57.8%, n=26). When trainees were asked about whether concerns about how the GMC will use reflective notes was a concern for them, 80.8% (n=160) of those who reflected said that it was, and 62.2% (n=28) of those who had not reflected also agreed that it was. However, there was only a small association between legal and GMC concerns and the likelihood of reflections (V=0.025 and V=0.173, respectively).

The factors, which had the strongest association with trainees' likelihood of writing reflections, were the location of primary medical qualification (p<0.001, V=0.687), the time of exposure to the concept of reflective practice (p<0.001, V=0.431), and whether or not they had ever received any training on reflection (p<0.001, V=0.3).

The entire group of trainees was asked to identify the single main barrier to their engagement with reflective writing in their portfolio and this is reflected in Table 3. The main perceived barrier to reflection was not having the time to do it for 46.9% (n=114) and not seeing any benefit to writing reflections for 31.7% (n=77).

**Table 3:** Trainees' main perceived barrier to reflection Data represented as %(n) N=Total population

Barriers	% (N=243)
No time	46.9 (114)
Don't see the benefit	31.7 (77)
GMC/Legal fears	10.7 (13)
Don’t know how	5.3 (13)
Multiple reasons	5.3 (13)

## Discussion

There is now a great expectation that clinicians will engage in reflection of some form throughout the entirety of their careers [9]. Physicians in any training program leading to consultant status are, reasonably, expected to develop this skill [10]. We have sought to understand whether all trainees record formal reflective notes in their e-portfolios in one of the largest training deaneries for internists in the UK. To our knowledge, there have not been any studies that have attempted quantitative analysis of this area among resident physicians, and we felt that understanding the scope of the problem is a first step to addressing the issue that is known to exist.

This study has found that there is a good level of engagement with written reflections in the trainees’ portfolios (81.5%, n=198) even though this is not a mandatory requirement. But equally, data also show that a significant number of trainees could potentially complete their three-year program without any evidence of engagement in formal reflection (Table 2). This helps to underscore the importance of this study which looks to quantify the barriers to reflective practice and address this level of non-engagement which could lead to missed opportunities for overall growth and pose challenges in accurately assessing their progress and readiness for independent practice, a matter of utmost importance in medical training [11].

There is variation in who is likely to write reflections by the stage of training with trainees further along in their program more likely to have written reflections (Table 2). This must be accepted cautiously given that the association between the stage of training and engagement in reflective practice is weak (V=0.19). Furthermore, the longer the trainees are in the program the higher the median number of reflective pieces in their portfolio (Table 1). This trend is supported by a 2017 paper where Winkle et al. suggest that there is a vertical component to reflective practice where reflection is increased and deepened by exposure [1]. They suggest that trainees in the later stages of their program may have had more time to become familiar with reflective practices and integrate them into their routines. And given that we also see that trainees with fewer years in clinical practice (not the same as the stage of training) are more likely to reflect we postulate that this represents a function of time rather than any significant change in trainees’ perspective throughout the program. That is to say that they have simply written more reflections because they have been in the program longer.

The study shows an overwhelming focus of the trainees’ reflective notes on clinical outcomes (positive and negative) as well as on their formal teaching events (Table 3). There is a suggestion in the literature that this is very likely a representation of the long-standing perception of clinicians that it is the diagnosis, treatment, and outcome of patients that are the real markers of clinical proficiency [12]. As such they do not tend to focus on patient encounters, communication challenges, and their attitudes and beliefs despite suggestions that these areas are among those that improve the most from reflective practice [1]. It also shows that within our context, the training program has not done much to sufficiently de-emphasize the clinician’s tendency to focus only on the scientific aspect of medicine. We suggest that the creation of forms within the e-portfolio with specific prompts to a wider range of areas for reflection may help to create an awareness of the value of reflection on all aspects of clinical practice.

We have seen that where trainees completed their primary medical qualification has a statistically significant impact on reflection and this association is very strong (Table 2, V=0.687). The stage at which trainees first became exposed to reflective practice and whether they were tutored/trained on how to reflect are other factors that are strongly associated (V=0.431 and V=0.3) with trainees engaging in reflective practice. It is clear from the data that the variation in the training background of the doctors who took this survey is by far the most important factor affecting their engagement in reflective practice. The finding is supported by the literature which talks about how the background of doctors is a foremost factor when considering the lack of engagement with reflection [13].

Trainees who underwent medical education in the UK were exposed to reflective practice in medical school and those who had been trained on how to reflect were far more likely to have engaged in reflective practice. This is supported by literature that suggests that most doctors from UK universities would encounter reflective practice from medical school [14]. The converse is also true, it is the doctors who are not trained in the UK and those who did not get exposed to reflective practice in medical school who are the least likely to write reflective notes. This is rather important given the context within which the clinician who took part in this survey exists. The National Health Service is a healthcare system with a very diverse demographic and its clinicians receive training all over the world [15]. There is a subset of trainees who may not have had prior exposure to reflective practice, especially if they only encountered the concept after entering medical practice in the UK. These individuals may require additional support and guidance to understand the purpose and benefits of reflective practice and to develop the skills necessary to effectively engage in it [16].

The findings from this study suggest that in our local context, one strategy could include educating doctors about all aspects of reflection at the onset of their training program as a means of increasing engagement with reflective practice. And even though, our data suggest that supervisor input has almost a negligible statistical effect on reflection (V=0.055). As suggested by Miller, supervisors do play a crucial role in facilitating reflection among trainees [17]. They can provide mentorship, guidance, and feedback on trainees' reflective writing, creating a supportive environment that encourages open discussion and self-reflection. Additionally, supervisors can incorporate reflective activities into clinical training sessions, debriefings, and one-on-one meetings with trainees which have great potential to act as the starting point for formal recorded reflection.

Finally, the numbers suggest that legal concerns and worries about how GMC can use reflective notes are statistically significant (Table 2). In the context of the UK, the case of Dr. Bawa-Garba likely explains this trend among the trainees [18]. The case raised significant questions about the role of reflective practice and its implications for legal proceedings and professional accountability. Doctors may worry that their reflective notes could be misinterpreted or used as evidence of incompetence, mistakes, or lapses in judgment. If there is clarity and/or assurances around this, then this could potentially again improve the level of engagement with portfolio-based reflective writing.

Limitations

The main limitation of this study is the fact that trainees who reflected were more likely to have been respondents in a survey asking about reflection. This form of bias is common with any survey-based study. Additionally, while our sample size may be representative of the region within which the survey takes place it would not be possible to extrapolate the findings to IMT across the entire UK. Therefore, the overall generalizability of our findings is limited. For future research, the mixed method is likely to add more to our understanding of the content of reflections as our data only gives us a small insight into this. Despite providing opportunities for open-ended responses in the questionnaires, trainees did not provide any. It seems likely that it was more convenient to choose from among the provided options. This may have affected the information provided.

## Conclusions

Addressing the issue of trainees' lack of engagement with formal reflection requires a multifaceted approach. Training doctors about reflection at the outset of their training program, coupled with ongoing support and guidance from supervisors, is essential for fostering a culture of reflective practice within medical education and ensuring that all trainees can engage meaningfully with reflection throughout their training and career.

## Appendices

**Table 4:** Questionnaire sent out to internal medicine trainees.

Questions	Responsed/Response Type						
1. What year of training are you currently in?	IMT1	IMT2	IMT3				
2.How many years has it been since you completed your primary medical qualification?	Open to whole number numerical values						
3.Where did you go to medical school?	UK	Europe	Australia	US&Canada	Middle East	Africa	Asia
4.What do you understand by the term reflective practice?	Open to short responses						
5. When were you first introduced to the concept of reflective practice in medicine?	Medical school	Overseas medical practice	UK medical practice	Unfamilar with concept	Other		
6. Written reflections are a component of the JRCPTB portfolio, have you engaged in writing any?	Yes	No					
7. How many reflections would you say that you have written in your portfolio up to this point in your training?	Open to whole number numerical values						
8.If yes, what have you reflected on?	Negative Clinical Outcome	Positive Clinical Outcome	Organized Teaching	Any new learning point	Other things, specify		
9. Are you worried your reflection could be used by the GMC in fitness to practice hearings?	Yes	No					
10. Have you ever decided not reflect because you were worried it could be used against you legally?	Yes	No					
11. Have you received any formal tutoring on the importance of reflection/how to reflect?	Yes	No					
12. Has any discussion with your ES/CS led to your writing a portfolio reflection?	Yes	No					
13. Have your ES/CS ever given feedback/asked to discuss any reflection you have written in your portfolio?	Yes	No					
14.Writing reflections in my portfolio has improved my clinical practice. To what extent do you agree?	1 (Strongly Disagree)	2	3	4	5 (Stronly Agree)		
15. What would you say is the single main barrier that stops you from writing reflections?	No time to do it	Don't know how	Don't see any benefit doing it	Fears about GMC/Legal use			
